# Multi-Faceted Systems Biology Approaches Present a Cellular Landscape of Phenolic Compound Inhibition in *Saccharomyces cerevisiae*

**DOI:** 10.3389/fbioe.2020.539902

**Published:** 2020-10-14

**Authors:** Eugene Fletcher, Kristin Baetz

**Affiliations:** Ottawa Institute of Systems Biology, Department of Biochemistry, Microbiology and Immunology, University of Ottawa, Ottawa, ON, Canada

**Keywords:** systems biology, synthetic biology, yeast, phenolic inhibitors, fermentation, metabolism, biomanufacturing

## Abstract

Synthetic biology has played a major role in engineering microbial cell factories to convert plant biomass (lignocellulose) to fuels and bioproducts by fermentation. However, the final product yield is limited by inhibition of microbial growth and fermentation by toxic phenolic compounds generated during lignocellulosic pre-treatment and hydrolysis. Advances in the development of systems biology technologies (genomics, transcriptomics, proteomics, metabolomics) have rapidly resulted in large datasets which are necessary to obtain a holistic understanding of complex biological processes underlying phenolic compound toxicity. Here, we review and compare different systems biology tools that have been utilized to identify molecular mechanisms that modulate phenolic compound toxicity in *Saccharomyces cerevisiae.* By focusing on and comparing functional genomics and transcriptomics approaches we identify common mechanisms potentially underlying phenolic toxicity. Additionally, we discuss possible ways by which integration of data obtained across multiple unbiased approaches can result in new avenues to develop yeast strains with a significant improvement in tolerance to phenolic fermentation inhibitors.

## Introduction

Biomanufacturing is transforming how new and existing platform chemicals are made in a way that is environmentally friendly, renewable, and sustainable. To make bio-derived chemicals competitive to fossil-derived chemicals, high productivity and cost reduction are a major consideration. Therefore, there has been a growing interest in using cheap and readily available feedstocks, such as plant material (lignocellulose) obtained from agricultural and forestry wastes.

Lignocellulose is an abundant and ubiquitous biomass feedstock that can be hydrolyzed to yield simple sugars which are fermented by yeast to produce bioethanol, fine chemicals, and other bioproducts (Abo Bodjui et al., 2019). However, converting lignocellulose to these products during biomanufacturing has its challenges. The sugars in lignocellulose exist as long polysaccharide chains in the form of cellulose and hemicellulose which are held together by lignin (Becker and Wittmann, 2019). In order to make the cellulose and hemicellulose polymers accessible for hydrolysis to release sugars for fermentation, a pre-treatment step is required to dissolve the lignin fibers holding the sugar polymers. While physical (pyrolysis), physicochemical (ammonia fiber explosion) and biological methods exist for pre-treating lignocellulose, chemical pre-treatment methods are commonly used since they are simple and efficient (Becker and Wittmann, 2019). Chemical pre-treatment involves the use of dilute acid or alkali to break down the lignin. As a result, phenolic compounds which are monomeric subunits of lignin are produced during the pre-treatment step (Palmqvist and Hahn-Hägerdal, 2000). Phenolic compounds inhibit enzymes used to hydrolyze cellulose (Qin et al., 2016) and in effect, limit the amount of sugars available for fermentation. Phenolics are also extremely toxic to yeast even in minute quantities and significantly inhibit yeast growth and fermentation (Ando et al., 1986; Adeboye et al., 2014) thus, reducing the product yield and increasing the cost of fermentation.

Phenolic compounds exist in different forms in lignocellulosic hydrolysates as phenolic acids (e.g., ferulic acid), phenolic aldehydes (e.g., vanillin), phenolic ketones (e.g., 4-hydroxyacetophenone), and phenolic alcohols. The concentrations of each of these compounds in hydrolysates vary depending on the plant material and the pre-treatment method used. They appear to have different toxic effects on the cell with phenolic aldehydes being the most toxic and completely inhibit yeast growth at concentrations as low as 1 and 5 mM for coniferyl aldehyde and vanillin, respectively (Adeboye et al., 2014). The different levels of toxicity of multiple phenolic compounds were confirmed in a study which showed that the chemical nature of phenolic compounds determine their toxicity and the physiological impact they have on the cell (Adeboye et al., 2014). This study was backed by another report that demonstrated that ferulic acid and coniferyl aldehyde, though structurally similar with the only difference being the functional group, presented very distinct chemogenomic profiles and inhibited yeast growth using specific mechanisms (Fletcher et al., 2019).

Apart from converting lignocellulosic materials to bioethanol and other chemicals by fermentation, there has been a recent interest in valorizing lignin in lignocellulose to produce precursors and final products for the fine chemicals industry (Becker and Wittmann, 2019; Li et al., 2019; Ponnusamy et al., 2019). Vanillin is an example of a valuable phenolic compound in the fine chemicals industry mainly used as flavor or scent in food, pharmaceuticals and cosmetics (Luziatelli et al., 2019). While a process has been developed for fermenting glucose to vanillin (Brochado et al., 2010), ferulic acid is an important precursor which can be converted to vanillin by engineering microbial cell factories to express feruloyl-CoA synthase and feruloyl-CoA hydratase (Luziatelli et al., 2019). Other phenolic compounds, such as eugenol present in grains and cereals can be converted to ferulic acid and subsequently to vanillin (Overhage et al., 2002; Di Gioia et al., 2009). Again, a major limitation of using engineered yeasts for ferulic acid conversion to vanillin is the issue of toxicity of both vanillin and its ferulic acid precursor. It is possible to remove phenolic compounds from lignocellulosic hydrolysates as they form to prevent toxicity to the yeast cells (Carter et al., 2011; Xue et al., 2018) but this comes at an extra manufacturing cost. Therefore, to cost-effectively achieve high yields of bioethanol and other bioproducts from lignocellulose by fermentation, there is the need to improve tolerance to phenolic fermentation inhibitors in yeast cell factories that are used for the bioconversion.

A thorough understanding of the mechanisms that modulate phenolic compound toxicity is required to engineer yeast strains that are tolerant to individual phenolic compounds and/or a complex mix of phenolics found in hydrolysates. As inhibitor tolerance is a multigenic complex trait (de Witt et al., 2019) global cellular approaches are required to identify key determinants associated with phenolic compound tolerance. Advances in systems biology approaches have revolutionized our ability to assess how cells respond to phenolic toxicity. The use of genome-wide approaches have given insight into how the cell responds to individual phenolics and identified genetic and metabolic targets that can be engineered to improve tolerance to toxic phenolic fermentation inhibitors. However, a comprehensive understanding of the phenolic tolerance pathway remains lacking since data from the individual studies have not been fully integrated.

Here, we review several unbiased functional genomics and transcriptomic approaches to identify general and specific genetic targets that modulate phenolic compound toxicity in *S. cerevisiae*. We also highlight the potential of exploiting proteomics and metabolomics approaches, which remain underutilized in the field. Finally, synthetic biology approaches and future developments that can rapidly be used to generate yeast tolerant to phenolic fermentation inhibitors are discussed.

## Functional Genomic Approaches

Improving yeast tolerance to phenolic compounds first requires the identification of genes and pathways that can be engineered to confer increased tolerance. Therefore, functional genomic tools including chemogenomic screens, adaptive laboratory evolution, genome shuffling, and high content imaging can be exploited to discover genes associated with biological processes underlying phenolic tolerance in yeast.

### Chemical Genomics

The availability of both haploid and diploid deletion mutant collections, in which the majority of yeast open reading frames have been systematically deleted has made it possible to conduct chemical profiling or chemogenomic screens (reviewed in Giaever and Nislow, 2014). In agar-based array screens, the deletion mutant library is pinned onto solid media containing sub-lethal concentrations of the compound(s) being tested. Following incubation, colony sizes of the mutant strains on the selection plates vs. control plates are quantified to obtain fitness scores for all the mutants. Mutants that lack genes required for growth in the presence of the compound show a significant growth defect and are hypersensitive to the compound. Mutants that are sensitive to a compound aid in the identification of proteins and pathways needed for survival upon exposure to inhibitors. On the other hand, mutants that grow better than the wild type in the presence of the compound are called “suppressors.” Though it is harder to generalize the mechanism(s) by which suppressors when deleted confer protection to a compound, one possibility is the suppressor protein increases toxicity of the compounds. An example of a suppressor gene is *BNA7* which was found to enable yeast growth, when deleted, in media containing ferulic acid (Fletcher et al., 2019). Interestingly, though Bna7 has a well-established role in the tryptophan catabolic pathway, no other components of this pathway when deleted conferred improved tolerance to ferulic acid.

**FIGURE 1:**
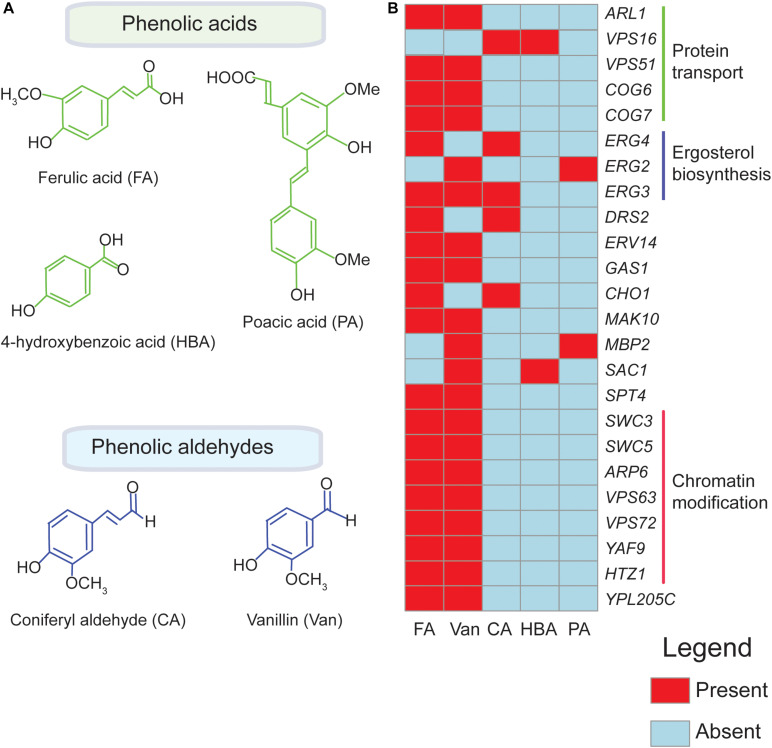
Genetic hits for different phenolics in functional genomics screens overlap. Chemical structures of phenolic compounds ferulic acid (FA), poacic acid (PA), coniferyl aldehyde (CA), 4-hydroxybenzoic acid (HBA), and vanillin (Van.) are shown in **(A)**. Overlapping biological processes of the deletion mutants identified in chemogenomic screens for FA, PA, CA, HBA, and Van are indicated in **(B)** The heatmap shown here is based on published data obtained from Endo et al. (2008), Skerker et al. (2013), Pereira et al. (2014), Piotrowski et al. (2015), Wang X. et al. (2017), Wu et al. (2017), Biot-Pelletier et al. (2018), Sardi et al. (2018), Xue et al. (2018), Fletcher et al. (2019), and Hacısalihoǧlu et al. (2019). The genes were clustered according to their associated biological process (Robinson et al., 2002).

Presently, four phenolics (coniferyl aldehyde, ferulic acid, 4-hydroxybenzoic acid, and vanillin) (Endo et al., 2008; Fletcher et al., 2019) have been screened through agar based methods to identify their chemogenomic profiles (Figure 1A and Table 1). The highest overlap in chemogenomic profiles (or shared gene “hits”) was found between ferulic acid and vanillin with 17 common deletion mutant genes with hypersensitivity shared between these two compounds, which is not unexpected considering vanillin is derived from ferulic acid. Most of these common genes clustered into biological processes [according to Gene Ontology enrichment analysis (Robinson et al., 2002)] mainly associated with protein transport (*COG6*, *COG7*, and *ARL1*), chromatin modification and transcription (*SWC3, ARP6, YAF9*, *HTZ1*) (Figure 1B; Endo et al., 2008; Fletcher et al., 2019). These biological processes serve as interesting targets for engineering a vanillin-producing yeast strain that uses ferulic acid as a precursor since tolerance to both phenolics will be required in such a strain. Also, wheat straw hydrolysate and synthetic miscanthus hydrolysate which contain a complex mixture of several phenolics has been screened for yeast tolerance (Skerker et al., 2013; Pereira et al., 2014; Table 1). Genes involved in protein synthesis (*RPL13B, RPL13A*), ergosterol biosynthesis (*ERG2*) and oxidative stress response (*SOD1, SOD2*) were among the top hits that came up in the screen (Skerker et al., 2013; Pereira et al., 2014). Since the yeast libraries contain over 4,000 non-essential mutants, the use of robotics in performing genome-wide screens is now taking center stage as it allows the screens to be performed rapidly and simply.

**TABLE 1:** Functional genomic strategies to elucidate yeast tolerance to phenolic inhibitors.

Screening tool	Fermentation inhibitor	Significant biological processes	References
Chemogenomic screen (agar-based array)	Ferulic acid	Protein and vacuolar trafficking Ergosterol biosynthesis	Fletcher et al., 2019
Chemogenomic screen (agar-based array)	4-hydroxybenzoic acid	Ergosterol biosynthesis Protein trafficking	Fletcher et al., 2019
Chemogenomic screen (agar-based array)	Coniferyl aldehyde	Pentose phosphate pathway	Fletcher et al., 2019
Chemogenomic screen (agar-based array)	Vanillin	Ergosterol biosynthesis Histone exchange	Endo et al., 2008
Chemogenomic screen (agar-based array)	Synthetic miscanthus hydrolysate	Ergosterol biosynthesis Oxidative stress response Pentose phosphate pathway	Skerker et al., 2013
Chemogenomic screen (well-by-well array)	Wheat straw hydrolysate	Vacuolar acidification Ribosome biogenesis Mitochondrial and peroxisomal function Ergosterol biosynthesis	Pereira et al., 2014
Chemogenomic screen (barcode sequencing of pooled mutants)	Poacic acid	Cell wall and glycosylation	Piotrowski et al., 2015
Chemogenomic screen (barcode sequencing of pooled mutants)	Synthetic corn stover hydrolysate	Fatty acid biosynthesis Vesicle trafficking	Xue et al., 2018
Adaptive laboratory evolution	Coniferyl aldehyde	Vacuolar transport Mitochondrial function Transcriptional regulation	Hacısalihoǧlu et al., 2019
Adaptive laboratory evolution	Vanillin	Transcriptional regulation	Wang H.-Y. et al., 2017; Wang X. et al., 2017
Genome shuffling	Lignocellulosic hydrolysate	Transcriptional regulation	Biot-Pelletier et al., 2018
Genome-wide association studies	Synthetic corn stover hydrolysate	Ergosterol biosynthesis Proteolysis	Sardi et al., 2018
Chemogenomic screen (well-by-well array)	Coniferyl aldehyde	Membrane transport Oxidative stress response	Wu et al., 2017

In addition, agar-based screens have also been used to perform focused screens. For example, a yeast deletion mutant array composed of 30 yeast transcription factor mutants has been screened to provide insight into phenolic-induced transcriptional changes. The study revealed that genes encoding the transcription factors *YAP1*, *DAL81, GZF3, LEU3, PUT3*, and *WAR1* were required by yeast to grow in coniferyl aldehyde (Wu et al., 2017). This approach provides preliminary information on transcription factors that can be engineered to concurrently regulate the expression of several genes associated with phenolic tolerance instead of engineering the individual genes they regulate. However, studies of this nature are limited by the size and composition of the mini-array being screened.

The yeast deletion libraries are barcoded with a unique 20 bp sequence placed upstream (uptag) and downstream (dntag) of the KanMX selection marker gene used to replace the gene of interest (Winzeler et al., 1999). Genetic barcoding is a powerful tool for even more complex fitness profiling of the mutant collection where thousands of yeast mutants are pooled, and grown in liquid media containing an inhibitor and analyzed in parallel (Shoemaker et al., 1996). Coupled to next generation sequencing (NGS), the amount of barcoded PCR product representing each mutant can be quantified to identify mutants with high tolerance to the inhibitor being tested (Smith et al., 2009). Genetic barcoding has the advantage of allowing screening of complex phenolic mixtures and plant hydrolysates (Skerker et al., 2013; Table 1). Furthermore, this method is useful for identifying suppressors since tolerant mutants outgrow the other strains in the mutant pool and are selected at the end of the experiment. For example, genetic barcoding was used to identify suppressor genes involved in fatty acid metabolism (*EEB1*) and vesicle trafficking (*SSH4* and *VAM6*) as important for conferring tolerance to a mixture of phenolics in a synthetic hydrolysate (Xue et al., 2018; Table 1). Similarly, by screening and sequencing a pooled yeast deletion mutant library, suppressor genes (*SUR1, NBP2, DFG1*) whose deletion resulted in tolerance to poacic acid were identified (Piotrowski et al., 2015; Table 1).

Although chemogenomic screens, serve as a powerful tool for identifying genes associated with phenolic compound tolerance (sensitive mutants), it remains a challenge to identify suppressors by either method. This is likely because in most cases the growth improvement of the suppressors is small at the sub-lethal concentrations these screens have been performed at. However, success at identifying suppressors can be improved by performing parallel chemical genomic screens at multiple dosages. Further, chemogenomic screens are limited in that they only screen the impact of loss of open reading frames, hence this type of screen excludes gain or separation of function mutations and mutations in regulatory regions. Again, current screens have only probed the non-essential genes, and have not probed the essential mutant collections. Plus, chemogenomic screens are not ideal for selecting tolerance phenotypes that are as a result of epistatic interactions between multiple genes since it only determines the effect of single-gene deletions or mutations.

To complement the chemogenomic method where yeast deletion libraries are screened, overexpression libraries, such as the MoBY collection (Hou, 2009) can be probed to identify genes whose overexpression result in tolerance to phenolic compounds. Even though this method has not yet been applied to phenolic tolerance, it has been demonstrated that by screening an overexpression library, a multi efflux pump, *SGE1*, was found to improve yeast tolerance to a yeast growth inhibitor used as a pre-treatment solvent (Higgins et al., 2018).

### Adaptive Laboratory Evolution

As an alternative approach, other studies have used adaptive laboratory evolution to point out key driver mutations that are essential to increase tolerance to phenolic compounds. In adaptive laboratory evolution, yeast cultures containing mild concentrations of the phenolic compound are serially transferred into fresh media supplemented with increasing concentrations of the phenolic compound until a significant improvement in growth rate is observed after several generations and serial transfers (Dragosits and Mattanovich, 2013). Advances in whole-genome sequencing technologies with regards to reduction in costs, new and improved sequence analysis tools (Sandmann et al., 2017) and sequencing platforms (Rhoads and Au, 2015; Tyler et al., 2018) have made it possible to identify mutations that lead to improved growth in the presence of phenolic compounds.

Adaptive mutations can occur in the regulatory regions or coding regions of the target gene and result in loss of function, increased activity, or decreased dosage of the gene product (Dragosits and Mattanovich, 2013). For example, the zinc finger transcription factor, *YRR1* acquired a frameshift mutation resulting in a loss of function which improved yeast growth in vanillin (Wang X. et al., 2017; Table 1). The role of *YRR1* in vanillin tolerance was confirmed by deleting the gene (Wang X. et al., 2017). In another example, nonsense mutations acquired by *MUK1* and *MRS4* resulted in tolerance to coniferyl aldehyde (Hacısalihoǧlu et al., 2019). The challenge with using adaptive laboratory evolution, though, is most times several mutations unrelated to the compound tolerance phenotype will arise making it challenging to pinpoint the actual mutations that are required for tolerance. It is possible to confirm each mutation gained in a laboratory evolution experiment but in instances where several mutations arise, a considerable amount of time is required to validate the effect of all the mutations in a wild type strain. One method to circumvent this challenge is to perform multiple parallel screens (Fletcher et al., 2017). High throughput evolution of several lines in parallel facilitated by robotics, automation and mutational analysis makes it possible to quickly identify common suppressors over parallel evolutions (Radek et al., 2017) but this has not yet been applied to phenolic screens.

### Genome-Shuffling

Laboratory evolution can be extended in another approach called genome shuffling to find novel genes that can be modulated for increased phenolic tolerance. Genome shuffling allows the discovery of positive epistasis and the accumulation of beneficial mutations. The technique involves performing mutagenesis on haploid yeasts of both mating types (a and α), selecting for tolerant haploids and mating them to obtain diploid strains (Hou, 2009; Pinel et al., 2011). The diploids are screened on media containing increasing concentrations of the phenolic compound after which the most tolerant diploids are isolated. The diploids with increased tolerance then undergo a new round of sporulation, mating and selection on increasing concentrations of phenolic compounds. Several cycles of “genome-shuffling” are performed to generate yeast strains with improved tolerance. The resulting strains are sequenced to identify key mutations that can then re-constructed in the wild type to confirm the role of the selected mutations on increasing tolerance. Using genome shuffling, genes including *NRG1*, *GSH1*, and *GDH1* were identified as key determinants required by yeast for improved tolerance to lignocellulosic hydrolysate (Biot-Pelletier et al., 2018; Table 1). Although genome shuffling has the advantage of filtering mutations that are unrelated to phenolic tolerance, it can be laborious and time consuming. Currently, the technology is challenged by the lack of high throughput screening methods (Magocha et al., 2018). As such, there is the need to automate the process to make it rapid and more efficient in identifying novel genetic mutations that are linked with improved tolerance to phenolic inhibitors in yeast.

### High Content Imaging

Another emerging technology is the use of a high content, image-based profiling to identify biological processes that are targeted by toxic compounds (Ohnuki et al., 2010). Here, it is assumed that changes in yeast morphology as a result of a chemical treatment will resemble the morphology of mutants that lack genes associated with biological processes that are inhibited by the chemical. High content imaging was used to identify genes associated with toxicity to vanillin by comparing the altered morphology of >4,000 yeast mutants to that of wild type yeast treated with vanillin (Iwaki et al., 2013). Using this technique, 18 mutants with an altered morphology that mimic the morphology of vanillin-treated cells were identified. Out of the 18 mutants, three mutants (*rpl8a*Δ, *rpp1b*Δ, and *rpl16a*Δ) that had the closest resemblance to vanillin-treated cells lacked genes belonging to the gene ontology (GO) term “cytoplasmic protein component of the large ribosomal subunit”(Iwaki et al., 2013). The outcome of the study indicates that vanillin toxicity may be due to inhibition of large ribosomal subunit leading to an impairment of protein synthesis.

Though useful in elucidating novel mechanisms of toxicity of phenolic compounds, a limitation of cell imaging is that it is not suitable for screening phenolic compounds that do not induce morphological changes. An extension of this technology will be the development of high throughput microscopy screening of the yeast GFP collection (Ghaemmaghami et al., 2003) to determine the impact of phenolic treatment on protein subcellular localization and abundance (Koh et al., 2015). For instance, upon coniferyl aldehyde treatment, several enzymes in the pentose phosphate pathway are both induced and partially localize to the mitochondria (Fletcher et al., 2019). It will be interesting to fully screen the yeast GFP collection to identify other proteins that change localization upon exposure to different phenolic compounds. Not only will such a screen provide an idea of what proteins are induced and change localization but it will also bring to light novel and alternative mechanisms of regulation where changes in protein localization render a pathway active or inactive due to unavailability of upstream intermediates.

## Transcriptomic Approaches

Exploring transcriptomic changes during exposure to phenolics provides another dimension to obtaining a holistic view of the phenolic tolerance landscape of yeast. Historically, microarray technology has been used to observe the expression of thousands of genes simultaneously to obtain a gene expression profile of cells under a given condition (Raghavachari, 2013). More recently, developments in high throughput RNA sequencing (RNA-Seq) has made it possible to quantify transcriptomes by measuring mRNA transcripts (Sardi et al., 2018). Unlike, microarrays which are limited by the genes included on the array, RNA-Seq allows for the identification of novel transcriptomic changes including alternative splice variants, novel genes and small mRNA sequences (Han et al., 2015).

Presently, two distinct transcriptomics approaches have been applied to unravel the biology of phenolic tolerance in yeast (Table 2). In one strategy, the transcriptome of phenolic-adapted strains are compared to that of un-adapted strains. Here, the goal is to identify the genes whose expression is modulated in the adapted strain as these genes and their associated biological pathways potentially confers tolerance to the phenolic inhibitor. In the second strategy, the transcriptome profile of yeast exposed to phenolic inhibitors are compared to that of an un-treated yeast. The goal of these experiments is to identify changes in gene expression upon phenolic exposure as these genes and their associated biological pathways may contribute to protecting the cell from phenolic toxicity.

**TABLE 2:** Transcriptomic profiling of yeast to elucidate cellular responses to phenolic inhibitors.

Screening tool	Fermentation inhibitor	Significant biological processes	References
Microarray analysis of a tolerant strain (non-stressed conditions)	Vanillin	Ergosterol biosynthesis Mitochondrial function	Endo et al., 2009
Microarray analysis of a tolerant strain (non-stressed conditions)	Coniferyl aldehyde	Oxido-reductase activity Oxidative stress response	Hacısalihoǧlu et al., 2019
Microarray analysis of a tolerant strain (non-stressed conditions)	Vanillin	Oxido-reductase activity Oxidative stress response	Shen et al., 2014
Microarray analysis of a tolerant strain (non-stressed conditions)	Vanillin	Response to stress Phospholipid metabolism	Wang X. et al., 2017
Microarray analysis of evolved strain (stressed conditions)	Softwood hydrolysate	Oxidative stress response Membrane transport	Thompson et al., 2016
Microarray analysis of wild type strain (stressed conditions)	Vanillin	TCA cycle Aerobic respiration	Park and Kim, 2014
Microarray analysis of wild type strain (stressed conditions)	Coniferyl aldehyde	Oxido-reductase activity Mitochondrial function	Sundström et al., 2010
Microarray analysis of wild type strain (stressed conditions)	Ferulic acid	Protein import Mitochondrial function	Sundström et al., 2010
Microarray analysis of wild type strain (stressed conditions)	Isoeugenol	Mitochondrial function	Sundström et al., 2010
Microarray analysis of *yrr1*Δ strain (stressed conditions)	Vanillin	Ribosome biogenesis rRNA processing	Wang X. et al., 2017

Remarkably, yeast strains evolved for vanillin tolerance in two independent studies displayed a similar transcriptome profile (Endo et al., 2008; Shen et al., 2014). Subunits of the cytochrome b-c1 complex (*QCR2, QCR10, QCR6*, and *CYT1*), which are components of the mitochondrial electron transport chain, together with an electron donor to the mitochondrial electron transport chain (*CYC1*), were upregulated in both vanillin-tolerant yeasts. This suggests an induction in aerobic respiration and energy generation in vanillin adapted strains is critical to confer tolerance (a summary of the transcriptome data comparing published transcriptome studies is provided in Supplementary Table 1). Furthermore, more than half of the upregulated genes that overlap in vanillin- (Endo et al., 2008; Shen et al., 2014) and coniferyl aldehyde- (Hacısalihoǧlu et al., 2019) adapted strains are involved in oxidation-reduction processes and NADPH production (*BDH2, CTT1, COX5B, SDH1, IDP2, CYB2, NDI1, ALD3, COX7, SPS19, ALD4*, and *ALD6*). Additionally, *FAA1, PRS3*, and *ALD5* were repressed in both vanillin-tolerant and coniferyl aldehyde-tolerant strains under non-stress conditions. The similarity in gene expression profiles in coniferyl aldehyde- and vanillin-adapted yeast from independent studies suggest that yeast utilizes common mechanisms to build tolerance to these compounds. Knowledge of these commonly induced pathways in adapted strains could be exploited to further improve phenolic tolerance.

Given the similarity in transcriptomes from adapted strains, it is somewhat surprising that transcriptome profiles of wild-type yeast exposed to phenolic compounds (ferulic acid, coniferyl aldehyde, vanillin, isoeugenol, and plant hydrolysates composed of a combination of these phenolic compounds) during growth share limited common features (Sundström et al., 2010; Park and Kim, 2014; Thompson et al., 2016). In these studies, apart from the mitochondrial potassium homeostasis gene *YLH47* that was upregulated in ferulic acid, coniferyl aldehyde and isoeugenol-treated cells, no particular set(s) of genes overlapped in all the studies. Though direct comparisons between transcriptomic studies have limitations (Larsson and Sandberg, 2006), these transcriptome studies suggests that the cell’s transcriptional response is largely distinct for each phenolic study so far.

Interestingly, four genes that are upregulated in the transcriptome of vanillin-adapted strains obtained under no stress (Shen et al., 2014) are also upregulated in the transcriptome of un-adapted yeasts treated with vanillin (Park and Kim, 2014). All four genes (*CIT1*, *LSC2*, *SDH1*, *SDH2*) encode enzymes in the TCA cycle. Similarly, genes involved in oxidation-reduction (*YML131W, YKL071W*, and *OYE3*), transport (*SNQ2*) and response to oxidative stress (*SRX1*) were upregulated in both coniferyl aldehyde-adapted yeasts (non-stressed conditions) (Hacısalihoǧlu et al., 2019) and in un-adapted yeasts treated with coniferyl aldehyde (Sundström et al., 2010).

Taken together, oxidation-reduction, electron transfer chain, and the TCA cycle are enriched in the transcriptome of yeast during phenolic toxicity suggesting an upregulation of mitochondrial activity during phenolic stress (Supplementary Table 1). A broader overview of transcriptome changes induced by phenolics in yeast studies are limited by the number of phenolics studied. There is the need to expand these studies to include a wide range of phenolic compounds to ascertain the effect of various phenolic compounds on the yeast transcriptome as a way of identifying potential biological processes that can be targeted to improve phenolic tolerance in yeast.

While transcriptomics can identify gene targets that when overexpressed or downregulated can improve phenolic tolerance, this technology is challenged by the fact that changes in the expression of most genes do not correlate with improved tolerance to yeast stress (Evans, 2015). Hence transcriptional profiling, while providing a holistic snapshot of the yeast’s response to a phenolic, may not provide a direct entry point into genetic engineering strains for phenolic tolerance improvement.

## Other Systems Biology Approaches

Though functional genomics and transcriptomic studies have so far dominated the field of yeast phenolic tolerance, they clearly do not capture all the biological events that occur upon exposure to phenolics. While functional genomics identify proteins and pathways required for survival upon exposure to phenolics, it fails to assess how these proteins are regulated and their biological role in phenolic tolerance. Gene expression modulation during phenolic compound stress serves as a tangible way of quantifying induction and repression of genes associated with phenolic toxicity. However, RNA levels can only be used as a proxy for measuring products of expressed genes within the cell and may not reflect protein levels, protein function or modification of proteins by post-translational modifications. Further, neither functional genomics nor transcriptomics can assess how phenolic exposure modifies a cell’s metabolism. Hence, two emerging systems biology approaches worth highlighting that can provide this extra layer of genome-wide information are proteomics and metabolomics.

## Proteomic Profiling of Yeast Phenolic Tolerance

### Shotgun Proteomics

One approach to capture protein changes in the cells is the shotgun proteomic method which involves digesting total cellular proteins (isolated from cells treated with or without a toxic compound) into peptides which are separated by liquid chromatography followed by identification and quantification using mass spectrometry (Zhang et al., 2013). Beyond identifying differential changes in protein expression, post-translational modification sites can be identified using quantitative methods, such as stable isotope labeling by/with amino acid in cell culture (SILAC) (Ong et al., 2004).

To date, very few studies have probed the proteomic profile of yeast upon phenolic exposure, using shotgun proteomics tools. A proteomic study quantified protein expression in two natural isolates of *S. cerevisiae* that exhibited remarkable tolerance to a synthetic inhibitor cocktail containing ferulic acid, cinnamic acid, and coniferyl aldehyde (de Witt et al., 2018). Their proteomic profile revealed a general tolerance mechanism which mainly included genes associated with oxido-reductase activity (de Witt et al., 2018). In another proteomic study, expression of oxidative stress response proteins (Ahp1 and Grx1) was found to be induced during yeast growth in a combination of three inhibitors which include phenol (Ding et al., 2012a). So far, the application of shotgun proteomics to understanding phenolic tolerance has been limited to quantifying protein expression. Future work should investigate post-translational modification of the most differentially expressed proteins during yeast growth in different phenolic compounds.

## Metabolomics Profiling of Phenolic Fermentation Inhibitors

Comprehensive analysis of metabolites during cellular stress is gaining popularity as another strategy to understand tolerance mechanisms (Nugroho et al., 2015). Therefore, metabolomics tools are being developed to obtain a cell’s metabolic profile or metabolome which directly reflects the cell’s metabolic state (Zampieri et al., 2017).

### Mass Spectrometry-Based Metabolomics

Developments in mass spectrometry-based metabolomics have enabled quantification of metabolites even at low concentrations with high resolution and dynamic range (Marshall and Powers, 2017). Extending this technology to understanding the underlying basis of phenolic toxicity in yeast, a metabolic shift between a parental yeast and an inhibitor-tolerant yeast was observed during growth in a mixture of inhibitors which included phenol (Ding et al., 2012b). The mixed inhibitors induced the production of myo-inositol and phenylamine in the tolerant yeast suggesting regulation of membrane trafficking and cytosolic Ca^2+^ concentration, respectively. Remarkably, glycolysis and TCA cycle intermediates including citrate, succinate, and 2-oxoglutarate were decreased in the tolerant strain during growth in the mixed inhibitors (Ding et al., 2012b). It will be interesting to further explore the effect of a wide range of phenolic compounds on changes in yeast metabolomic profiles using untargeted metabolomic tools followed by a targeted approach to confirm any observed metabolic shifts.

## Mining Genome-Wide Studies to Identify Common Approaches to Improve Tolerance to Phenolics

The different systems biology tools discussed above have highlighted several biological processes associated with phenolic tolerance. While proteomic and metabolomics data is currently limited for phenolics, the functional genomics and transcriptomics data can be used to identify common mechanisms underlying phenolic toxicity. By targeting common mechanisms it may be possible to engineer strains with improved tolerance toward all phenolic compounds. Four common cellular responses to phenolic exposure that have been identified from the functional genomics and transcriptomics data are: oxidative stress response, oxido-reductase and mitochondrial activity, ergosterol biosynthesis, and membrane transport.

### Oxidative Stress Response

Functional genomic screens determined that deletion of genes involved in oxidative stress response (*YAP1, STB5, GSH1, SOD1, SOD2*) resulted in hypersensitivity to phenolics (Skerker et al., 2013; Pereira et al., 2014; Wu et al., 2017; Biot-Pelletier et al., 2018). Also, *YAP1, SOD2* and other genes with antioxidant activity (*GRX2, CTT1, CTA1*) are upregulated during phenolic exposure as shown in the transcriptomic studies (Park and Kim, 2014; Shen et al., 2014). Furthermore, Grx1 and Ahp1 which are oxidative stress response genes are differentially enriched in proteomic studies when yeasts are treated with phenolics (Ding et al., 2012a) suggesting the production of reactive oxygen species (ROS). Together, this suggests that phenolic exposure elicits an oxidative stress response in yeast.

Experimental evidence shows that phenolic compounds, particularly those with an aldehyde functional group, such as vanillin and coniferyl aldehyde induce ROS formation (Nguyen et al., 2014; Fletcher et al., 2019). ROS production comes from a combination of aerobic respiration and possibly from the endogenous process of oxidizing the compound to less toxic forms (Adeboye et al., 2015; Hacısalihoǧlu et al., 2019). Yap1, a major oxidative stress transcription factor that regulates the expression of several genes responsible for scavenging ROS (Maeta et al., 2004; Rodrigues-Pousada et al., 2010) changes localization from the cytosol to the nucleus upon vanillin treatment where it activates transcription of its target genes (Nguyen et al., 2014). Not surprisingly, some genes regulated by Yap1 including *GPX1* and *SOD2* are significantly upregulated in a transcriptomic screen for vanillin (Shen et al., 2014). Additionally, yeast functional genomic study showed that *YAP1* deletion results in hypersensitivity to synthetic miscanthus hydrolysate which contains a mixture of phenolic compounds including coniferyl aldehyde (Skerker et al., 2013). Although it has been previously established that ferulic acid, 4-hydroxybenzoic acid and coniferyl aldehyde induce ROS formation in yeast, the specific species of ROS induced by different phenolic compounds is yet to be identified.

Catalases and the glutathione pathway which scavenge ROS from the cell require NADPH (Toledano et al., 2013; Gómez et al., 2019). Interestingly, not only are oxidative stress response proteins differentially expressed during treatment with phenolic inhibitors, as revealed in a proteomics screen, but also flux is moved toward the pentose phosphate pathway (Lv et al., 2014), one of the main metabolic pathways that generate cytosolic NADPH (Chen et al., 2019). Indeed, this proteomic study is corroborated by a functional genomic screen which showed that pentose phosphate pathway mutants accumulated ROS and were hypersensitive to coniferyl aldehyde (Fletcher et al., 2019). Moving forward, knowledge of the specific ROS species induced by individual phenolics will be beneficial in tailoring and engineering specific tolerance pathways for individual phenolic compounds.

### Oxido-Reductase and Mitochondrial Activity

Coniferyl aldehyde, vanillin, synthetic hydrolysates, and softwood hydrolysate induce an upregulation of genes involved in oxidoreductase activity and mitochondrial function (Endo et al., 2009; Sundström et al., 2010; Shen et al., 2014; Thompson et al., 2016). Also, functional genomic studies identified other proteins with mitochondrial activity (*MRS4* and *AFG3*) that play a role in phenolic tolerance (Fletcher et al., 2019; Hacısalihoǧlu et al., 2019). A proteomic screen identified several oxido-reductases including Adh7, Adh4, and Ald6 as the most differentially expressed proteins upon phenolic treatment (de Witt et al., 2018).

In the context of tolerance to phenolic inhibitors, the mitochondria are an important site for detoxification of phenolic compounds as enzymes, such as Ald5 and Pad1 that catabolize phenolic aldehydes are located in the mitochondria (Adeboye et al., 2017). Detoxification of phenolics is a strategy employed by cells to prevent intracellular accumulation. Conversion of vanillin and coniferyl aldehyde to less toxic compounds catalyzed by enzymes with an oxido-reductase activity is upregulated during exposure to these compounds (Wang H.-Y. et al., 2017; Hacısalihoǧlu et al., 2019). Furthermore, a gene encoding an NADH-dependent aldehyde reductase (*YLL056C*) is enriched in yeast exposed to coniferyl aldehyde (Sundström et al., 2010). While degradation of coniferyl aldehyde by Yll056c has not been reported, enzymatic reduction of other toxic aldehydes found in lignocelluloic hydrolysates (furfural and glycoaldehyde) has been demonstrated (Wang H.-Y. et al., 2017). Lastly, the NADPH-dependent alcohol dehydrogenase, Adh6, contributes to vanillin tolerance by converting intracellular amounts of vanillin to the less toxic vanillyl alcohol (Nguyen et al., 2015). Moving forward, it will be interesting to determine if mitochondrial function or content, for example increased mitochondrial volume per cell, can be engineered to improve phenolic tolerance.

### Ergosterol Biosynthesis

Ergosterol biosynthesis genes (*ERG5, ERG26, ERG7, HMG1*, *ERG28*) have been reported to upregulated upon phenolic exposure in transcriptomic studies (Endo et al., 2009; Sardi et al., 2016). Confirming the role of ergosterol biosynthesis in phenolic tolerance, several functional genomics studies have shown that deletion of ergosterol biosynthesis genes result in a growth defect in the presence of individual phenolics and plant hydrolysates (Endo et al., 2008; Skerker et al., 2013; Piotrowski et al., 2015; Fletcher et al., 2019).

These data suggest that maintaining the proper levels of ergosterol is essential for growth in the presence of phenolic compounds. Indeed, increased levels of ergosterol are seen in vanillin-tolerant yeasts (Endo et al., 2009; Zheng et al., 2017) further confirming the need for ergosterol in the cell during exposure to phenolic compounds. Cellular ergosterol plays different roles in cells mainly by maintaining membrane integrity (Abe and Hiraki, 2009) and acting as components of lipid rafts (Eisenkolb et al., 2002; Bastos et al., 2012) which are possibly required for proper cellular function during growth in phenolics. Fine-tuning ergosterol levels and spatial localization to organelles may offer a unique way to buffer the toxic effects of phenolics.

### Membrane Transport

Integration of systems biology tools reveal membrane efflux as another significant mechanism used by yeast as a survival strategy during growth in phenolic compounds. A functional genomic screen showed that the deletion of genes encoding membrane transporters, *PDR5, YOR1*, and *SNQ2* made *S. cerevisiae* sensitive to coniferyl aldehyde (Hacısalihoǧlu et al., 2019). Also, *SNQ2* and another transporter, *MCH2*, were upregulated in transcriptomic studies during yeast growth in vanillin (Park and Kim, 2014; Wang X. et al., 2017).

Enrichment of genes encoding membrane transporters during exposure to vanillin hints that flushing out phenolic compounds from the cell prevents intracellular accumulation (Thompson et al., 2016; Wang X. et al., 2017; Hacısalihoǧlu et al., 2019). Not surprisingly, Pdr1 which regulates the transcription of these transporters was identified during a screen of transcription factor mutants for coniferyl aldehyde tolerance (Wu et al., 2017). Export of phenolics from the cell complements efforts used by the cell to detoxify intracellular amounts of the compound. These transporters require ATP for activity (Katzmann et al., 1995; Mamnun et al., 2004). Hence, in order to meet the ATP needs of the cell, aerobic respiration *via* the TCA cycle and the mitochondrial electron transport chain are induced during growth in vanillin, coniferyl aldehyde and lignocellulosic hydrolysates as revealed in multiple studies (Endo et al., 2009; Park and Kim, 2014; Sardi et al., 2016; Thompson et al., 2016). Besides, since the transporters localize to the plasma membrane, changes in membrane composition and integrity have severe consequences on their activity (Kodedová and Sychrová, 2015). Not surprisingly, genes ascribed to fatty acid metabolism (*TES1*), ergosterol biosynthesis (*ERG5, ERG7, ERG26, HMG1, ERG28*) and cell membrane-associated proteins (*HES1, PUN1*) are upregulated during growth in phenolic compounds (Endo et al., 2009; Sardi et al., 2016; Thompson et al., 2016).

Taken together, in dealing with phenolic compound toxicity, it is evident that *S. cerevisiae* upregulates its oxido-reductase machinery to oxidize and/or reduce phenolic compounds into less toxic forms as well as deal with the oxidative stress associated with this conversion. In addition, the mitochondrial function is upregulated to ensure ATP production required for the activity of transporters which potentially extrudes the phenolic compounds and/or the detoxified forms of it.

## Metabolic Engineering Considerations and Synthetic Biology Tools to Improve *S. cerevisiae* Tolerance to Phenolic Fermentation Inhibitors During Biomanufacturing

ROS scavenging, regulation of ergosterol biosynthesis and compound efflux serve as general phenolic tolerance pathways that can be engineered in a yeast production strain. Beyond engineering a general tolerance pathway, more distinct genetic modifications can be incorporated to result in tolerance to particular phenolics. For instance, deleting *YRR1*, *MRS4*, and *BNA7* to specifically increase tolerance to vanillin (Wang X. et al., 2017), coniferyl aldehyde (Hacısalihoǧlu et al., 2019), and ferulic acid (Fletcher et al., 2019) respectively. To rapidly facilitate these metabolic engineering strategies in building a phenolic tolerance pathway, genome editing tools, such as the CRISPR/Cas technology will make this possible (Li et al., 2020).

With the CRISPR/Cas technology, stable genetic modifications including introduction of specific mutations can be inserted into both promoter regions and coding regions of genes. This will facilitate modulating the transcription of genes required for phenolic tolerance (Giersch and Finnigan, 2017). Alternatively, expression of target genes can be regulated using a repurposed CRISPR system referred to as CRISPR interference (CRISPRi) (Qi et al., 2013). Similarly, introduction of mutations into the coding region can target protein modification sites (Giersch and Finnigan, 2017), modify activity (gain or loss of function), or changes in localization. Other modifications possible with the CRISPR/Cas method are gene insertions and deletions (Akhmetov et al., 2018). Insertion of extra copies of genes is done to stably overexpress target genes whereas multiple gene deletions can be used to knock out specific metabolic pathways (Giersch and Finnigan, 2017). Presently, CRISPR/Cas technology has not yet been applied to improving phenolic tolerance in yeast although the technology has been used to improve tolerance to other fermentation-related stresses in yeast (Giersch and Finnigan, 2017).

While growth (biomass yield) and tolerance are closely connected, making certain genetic manipulations associated with phenolic tolerance may lead to unwanted trade-offs in production hosts which can negatively impact product yield and titers. Therefore, to ensure the success of engineering yeast phenolic tolerance, metabolic flux analysis should be performed to assess the effect of the genetic modifications on the general physiology of the cell as well as carbon flux toward product formation. Such metabolic flux analyses should quantify and guide efficient resource allocation to ensure that cellular resources, such as NADPH and ATP are not diverted to phenolic tolerance at the expense of biosynthesis of bioproducts in the production strain.

Lastly, in the context of producing phenolic compounds, such as vanillin by fermentation, metabolic regulation of pathways that result in the catabolism of the phenolic as a detoxification strategy should be eliminated to improve yields.

## Future Outlook and Concluding Remarks

Overall, different systems biology approaches have been used to track global phenolic stress responses in *S. cerevisiae*. While common themes or mechanisms coincide among multiple studies, the different approaches provide alternative pathways and biological processes that can be exploited for strain improvement. Moving forward, since most of the long list of genetic hits reported in the various studies has not been validated, significant effort is required to confirm their actual role in tolerance or sensitivity to phenolics. This is particularly crucial for the transcriptomics data because the fact that a gene is upregulated or enriched during stress does not necessarily mean an overexpression of that gene will result in tolerance (Evans, 2015). Gene enrichment could merely be a stress response and not a tolerance mechanism. If possible, the role of enriched genes associated with phenolic tolerance should be confirmed by deleting and/or overexpressing target genes in cells grown in the presence phenolic inhibitors. Such confirmed genes should be cataloged in a “phenolic stressome” database similar to the yStreX (Wanichthanarak et al., 2014) as a repository where synthetic biologists can search for genetic targets to engineer tolerance to different phenolic compounds. By applying synthetic biology tools, such as the CRISPR/Cas technology, the expression of single or multiple genes identified in the “phenolic stressome” can be regulated in order to improve tolerance to phenolic compounds.

Finally, establishing the metabolomic profile of *S. cerevisiae* that are tolerant to a wide spectrum of individual phenolics may guide the development of biosensors to detect “signature metabolites” characteristic of tolerant and high-performing strains. Again, using synthetic biology, biosensors can be constructed with promoters (that are responsive to metabolites characteristic to tolerant and high-performing strains) and a reporting system (e.g., GFP), and inserted into a library of yeast mutants. Next, by applying microfluidics, a pool of heterogenous yeast mutants can be sorted to isolate phenolic tolerant strains that can be used in fermentation-based biomanufacturing to increase product yield and titers.

## Author Contributions

EF and KB conceived and designed the study. EF drafted the initial manuscript. KB edited and corrected the manuscript. Both authors contributed to the article and approved the submitted version.

## Conflict of Interest

The authors declare that the research was conducted in the absence of any commercial or financial relationships that could be construed as a potential conflict of interest.
